# Curcumin attenuates gentamicin and sodium salicylate ototoxic effects by modulating the nuclear factor-kappaB and apoptotic pathways in rats

**DOI:** 10.1007/s11356-022-21932-1

**Published:** 2022-07-20

**Authors:** Yasmina M. Abd-Elhakim, Sabry M. Abdel-Motal, Seham M. Malhat, Hend I. Mostafa, Walied M. Ibrahim, Rasha R. Beheiry, Attia A.A. Moselhy, Enas N. Said

**Affiliations:** 1 Department of Forensic Medicine and Toxicology, Faculty of Veterinary Medicine, Zagazig University, Zagazig, Egypt; 2 Department of Pharmacology, Faculty of Veterinary Medicine, Zagazig University, Zagazig, Egypt; 3 Department of Pharmacology, Animal health research institute, Zagazig, Egypt; 4 Audiology unit, Otorhinolaryngology Department, Faculty of Medicine, Zagazig University, Zagazig, Egypt; 5 Department of Histology and Cytology, Faculty of Veterinary Medicine, Zagazig University, Zagazig, Egypt; 6 Department of Anatomy and Embryology, Faculty of Veterinary Medicine, Zagazig University, Zagazig, Egypt; 7 Department of Veterinary Public Health, Faculty of Veterinary Medicine, Zagazig University, Zagazig, Egypt

**Keywords:** Gentamicin, Sodium salicylate, Curcumin, Ototoxicity, Apoptosis, NF-κB

## Abstract

This study aimed to investigate the effectiveness of curcumin (CCM) against gentamicin (GEN) and sodium salicylates (NaS)-induced ototoxic effects in rats. For 15 consecutive days, seven rat groups were given 1 mL/rat physiological saline orally, 1 mL/rat olive oil orally, 50 mg/kg bwt CCM orally, 120 mg/kg bwt GEN intraperitoneally, 300 mg/kg bwt NaS intraperitoneally, CCM+GEN, or CCM+NaS. The distortion product otoacoustic emission measurements were conducted. The rats’ hearing function and balance have been behaviorally assessed using auditory startle response, Preyer reflex, and beam balance scale tests. The serum lipid peroxidation and oxidative stress biomarkers have been measured. Immunohistochemical investigations of the apoptotic marker caspase-3 and the inflammatory indicator nuclear factor kappa (NF-κB) in cochlear tissues were conducted. GEN and NaS exposure resulted in deficit hearing and impaired ability to retain balance. GEN and NaS exposure significantly decreased the reduced glutathione level and catalase activity but increased malondialdehyde content. GEN and NaS exposure evoked pathological alterations in cochlear and vestibular tissues and increased caspase-3 and NF-κB immunoexpression. CCM significantly counteracted the GEN and NaS injurious effects. These outcomes concluded that CCM could be a naturally efficient therapeutic agent against GEN and NaS-associated ototoxic side effects.

**Graphical abstract:**

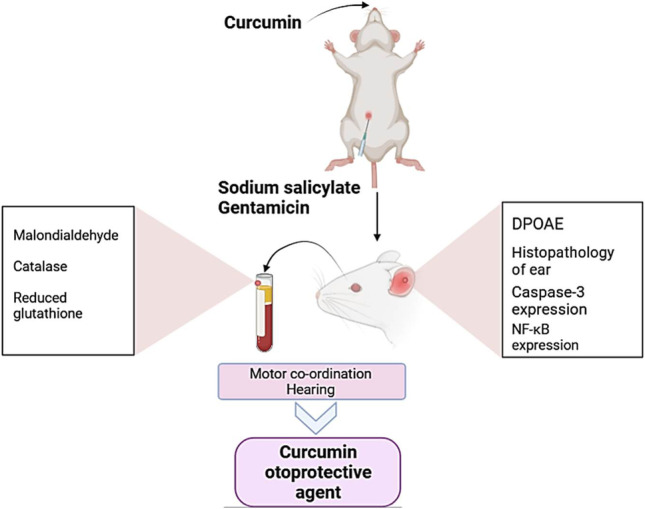

## Introduction

Certain medications are likely to cause cochlear and/or vestibular system cell degeneration, resulting in temporary or irreversible hearing loss, ataxia, tinnitus, dizziness, ear infections, nystagmus, vertigo hyperacusis, and other ear problems (Kim et al. 2022; Tang et al. 2021). The precise ototoxicity assessment has become a significant challenge for pharmacologists and toxicologists (Zhang et al. 2020). Antibiotics and salicylates are among the most frequently used pharmaceuticals in inpatient and outpatient settings (Friedrich 2018; Karalis et al. 2020; Van Boeckel et al. 2014).

Gentamicin (GEN) is an aminoglycoside antibiotic highly consumed for managing many bacterial infections. It is also the main first-line medicinal drug for neonates’ early infections (Johnson and Messier 2016; Karahan et al. 2005). The main side effects of GEN use are ototoxicity and nephrotoxicity (Elsakka et al. 2020; Koçak et al. 2017). Sodium salicylate (NaS) is a popular anti-inflammatory and pain reliever (Wang et al. 2008). Tinnitus is the main NaS overdose complication in human patients (Cazals 2000) and laboratory animals (Stolzberg et al. 2012). However, GEN and NaS remain widely used, mainly in developing countries, as they are cost-effective and not subject to strict regulations by prescription (Park et al. 2017). Developing otoprotective strategies is a key and urgent priority to avoid ototoxicity triggered by GEN or NaS.

Previous studies have verified the GEN and salicylate toxicity association with oxidative stress (Ansari et al. 2016; Bustos et al. 2018; Mohamed et al. 2019b). Furthermore, enhanced reactive oxygen species (ROS) production after aminoglycoside treatment has been shown to promote apoptotic hair cell death via caspase-3 and nuclear factor kappa (NF-κB) activation (Lee et al. 2004). NF-κB encompasses an inducible transcription factors family that regulates host inflammatory responses (Yamamoto et al. 2009). In particular, for normal hair-cell functions, comprising homeostasis of Ca^2+^, the family NF-κB is necessary, and signaling may react quickly to ototoxic stimulants to protect the hair cells and spiral ganglion cells (Jiang et al. 2005; Nagashima et al. 2007).

There is a global trend toward employing natural supplements to protect against drug-related side effects (El-Rahman et al. 2020; Hashem et al. 2020; Mohamed et al. 2019b). Specifically, many in vivo studies revealed that natural antioxidants could effectively protect against drug-induced ototoxicity (Aksoy et al. 2015; Fetoni et al. 2015; Heinrich et al. 2008). Curcumin (CCM), the *Curcuma longa* Linn yellow pigment, is a potent inhibitor of ROS formation (Biswas et al. 2005). As a result, CCM showed beneficial roles in a variety of health problems such as autoimmune diseases (Aggarwal and Harikumar 2009), cancer (Momtazi and Sahebkar 2016), diabetes mellitus (Pivari et al. 2019), and liver disease (Abd-Elhakim et al. 2021c; Jalali et al. 2020). Also, CCM has strong anti-inflammatory (Aggarwal and Harikumar 2009), antiapoptotic (Abd-Elhakim et al. 2021b), antigenotoxic (Saber et al. 2019), and immunostimulant (Mollazadeh et al. 2019) activities. Besides, CCM has shown important protective effects against widespread ototoxic substances and hearing disorders (Bucak et al. 2015; Soyalıç et al. 2017; Soyalıç et al. 2016). In addition, CCM protected cochlear fibroblasts from diabetes-related oxidative damage in rat models (Haryuna et al. 2017). Moreover, CCM decreased the apoptotic index in the cochlea lateral wall in ototoxic rat models produced by intratympanic injection of GEN for 18 h (Haryuna et al. 2018). Furthermore, the downregulation of NF-κB expression in the main cochlear structures has been proposed as underlying mechanism of the protective effect of CCM against cisplatin-induced ototoxicity (Paciello et al. 2020).

Based on the mechanism of action of both GEN and NaS and the earlier reported biological activity of CCM, we hypothesized that antioxidants like CCM could alleviate GEN or NaS ototoxicity. Hence, in the current study, rats were exposed to CCM and/or GEN or NaS for consecutive 15 days then subjected to distortion product otoacoustic emission (DPOAE) measurements and behavioral evaluation of the hearing and balance. Besides, biochemical, pathological, and immunohistochemical assessments were performed to evaluate if CCM could afford therapeutic effectiveness against GEN or NaS accompanied ototoxic effect.

## Material and methods

### Test compounds

GEN (garamycin; 80 mg/2 mL) was obtained from Memphis Co. & Chem. Ind., Cairo, Egypt. CCM (C_21_H_20_O_6_; 97% purity) was purchased from Sigma Company (St. Louis, MO, USA). Olive oil (Colavita, Rome, Italy) was used to prepare a CCM stock solution. NaS was obtained from El Nasr pharmaceutical chemicals “Adwic,” Cairo, Egypt. Ketamine (Ketalar) was obtained from Pfizer, New York, USA. Xylazine (Rompun) was obtained from, Bayer, Leverkusen, Germany. All other chemicals were obtained from Sigma-Aldrich Co. St. Louis, MO, USA.

### Animals and experimental design

Sprague–Dawley rats (male, 220–250 g, 12 weeks old) were obtained from the Animal Housing Unit, Faculty of Veterinary Medicine, Zagazig University, Egypt. The rats were housed in a stainless steel cage with free accessible food and water in an air-conditioned room in a 12-h light/12-h dark cycle. To ensure normal hearing of all rats included in the experiment, the external ear canals and tympanic membranes of all rats were examined using an operating ear microscope. Also, DPOAE measurements were performed. The criteria for exclusion were as follows: rats with signs of external ear disorders (impacted earwax, tumors, external acoustic meatus edema, and hyperemia), rats with middle ear disorders (opacification, hyperemia, and bulging or perforations of the tympanic membrane), and rats without DPOAE in any of the studied frequencies (1–8 kHz). All efforts were adopted to handle the rats humanely and achieve ethical rules throughout the experiment.

Rats were allowed to adapt to laboratory conditions 2 weeks before initiating the studies. Seventy rats were distributed randomly into seven groups (10 rats/group). Physiological saline (1 mL/rat) was administered orally to the control group. Olive oil (1 mL/rat) was administered orally to the solvent control group. CCM in 1 mL OO (50 mg/kg body weight) was administered to the test protective agent group; CCM was dissolved in OO (Akintunde et al. 2019). GEN (120 mg/kg bwt in 1 mL saline) was administered i.p. to GEN positive control group (Somdaş et al. 2015). NaS (300 mg/kg bwt in 1 mL saline) was administered i.p. to NaS positive control group (Chen et al. 2010). CCM+GEN were administered as above to the GEN experimental group. CCM+NaS were administered as above to the NaS experimental group. Rat received all test compounds for consecutive 15 days.

### Dose and route selection of GEN, NaS, and CCM

In the current study, GEN was intraperitoneally injected at 120 mg/kg bwt This dose has been chosen based on several earlier studies that confirmed the GEN ototoxic effect at this dose, route, and duration of exposure (Sagit et al. 2015; Sagit et al. 2014). Moreover, Somdaş et al. (2015) performed a preliminary study testing different doses of GEN (80–120 mg/kg bwt). They established the dose of 120 mg/kg bwt for the GEN-induced ototoxicity in a rat model. High doses of NaS have been reported to elevate neural thresholds and broaden auditory filters (Cazals 2000; Chen et al. 2010). In addition, Lobarinas et al. (2006) tested a range of doses of Nas (150–300 mg/kg bwt) and established the dose of 300 mg/kg bwt NaS intraperitoneally injected, as in our case, as a model of tinnitus in rats. The dose of 50 mg CCM /kg bwt has been reported to have antiapoptotic, antioxidant, and anti-inflammatory effects on various body organs in rats (Guo et al. 2020; Yang et al. 2019; Zbarsky et al. 2005). Moreover, the tested dose of CUR showed neuroprotective activity when orally given to rats for 15 days (Akintunde et al. 2019) but has not been tested before for otoprotective effects.

### DPOAE measurements

On the day of the last dosing in the experiment (15th day), rats were anesthetized by intraperitoneal injection of ketamine (50 mg/kg bwt) and xylazine (5 mg/kg bwt). DPOAE measurements were carried out in a silent room using the Otodynamic ILO-288 Echoport device (Otodynamics Ltd., London, UK). A suitable plastic tube adaptor (1 cm) was inserted into an external auditory canal with a plastic tympanometer probe. For DP-gram measurements, the primary stimulus values were equalized to 80 dB (L1, L2 = 80 dB sound pressure level (SPL)). In addition, two distinct frequencies (f1 and f2) are set to the 1.22 f1/f2 ratio to achieve optimal responses. DP gram measurements were carried out at frequencies of 1 to 8 kHz. DPOAE results were presented as the values above the noise floor at each frequency.

### Behavioral analysis

In the same examination room, all behavioral assessments have been performed. The treatments of the various experimental groups were unknown to the observer. On test days, rats were transported in their cages to the test room and allowed to adjust for 30 min before testing. At the end of each test, the rats were returned to their home cage, and the device was washed with a moist sponge to remove any odor.

#### Beam balance scale

Motor coordination was assessed using the beam balance test (Luong et al. 2011). Rats were positioned in the middle of an overall beam 2 m long, 1.5 cm wide, and 50 cm high above the floor. For 3 days, animals had been conditioned four times a day. The time without slipping (up to 60 s) on the balancing beam was documented three times in the test steps, and the obtained values were averaged. The rat’s performance was graded as 1 = stable; 2 = shaky balance; 3 = hugs beam, slips, hangs; 4 = falls after 10 s; 5 = falls before10 s; 6 = falls off with no attempt to balance.

#### Preyer reflex

According to Jero et al. (2001), a motion-tracking camera system was used to observe the pinna flexion. The motion-tracking system is comprised of four infrared cameras. Every pinna was fitted with a reflection marker (4 mm in diameter). An additional marker to a central point was fastened to determine the animal’s orientation, normally at the back center. The motion-tracking device used those markers to triangulate the ears’ location, accompanied by positive or negative pinna movement.

#### Auditory startle response

The hearing was assessed by placing the rat on a table and observing the response to handclaps and sharp metallic sounds (Koch and Schnitzler 1997).

### Sampling

On the termination of the dosing, rats have fasted overnight. All rats were weighed and anesthetized with ketamine/xylazine. The blood samples were collected from the retroorbital plexus of rats and centrifuged at 3000 rpm for 10 min. The produced serum was collected and kept at 20 °C until biochemically analyzed. The rats were euthanized by decapitation, then dissecting the cochlea and vestibular apparatus. The specimens were preserved in 10% neutral buffered formalin for histopathological and immunohistochemical examinations**.**

### Evaluation of serum oxidative stress and lipid peroxidation indicators

The Ohkawa et al. (1979) technique was used to determine malondialdehyde (MDA) content. In addition, catalase (CAT) enzyme levels were measured following the procedures of Aebi (1984), and reduced glutathione (GSH) estimations were made via the protocol described by Beutler et al. (1963)

### Histopathological investigations

The cochleae specimens were carefully separated from the temporal bone immediately after decapitation and washed with water. A small hole was created on the cochlear capsule’s apex, and the fixation was carefully forced with a thin needle to penetrate the cochlea completely by a fixative. After softening in EDTA decalcification solution, specimens were washed in running tap water and processed for paraffin blocks. Serial sections (5 μm thick) were cut and subjected to H&E stain (Suvarna et al. 2018). Five non-repeated randomly selected microscopic fields (40×) were examined in three different slides from each animal/group for lesion scoring. ImageJ software (http://Sb.Info.nih.gov/ij/) was used for performing the quantitative measurements, including the spiral ganglion cells and the number of hair cells at a 40× magnification.

The mean values in the examined five microscopic fields were considered the final lesion score per animal. The cochlea’s reported histopathological lesions in all groups were scored as follows: no change = zero, mild change = 1, mild to moderate change = 2, moderate change = 3, moderate to severe change = 4, severe change = 5.

### Caspase-3 and NF-κB immunohistochemical investigation in the cochlear tissues

Another group of cochlear paraffin sections was used for caspase-3 detection by a rabbit polyclonal antibody (cat. no. RB-1197-R7 Thermo Fisher Scientific, Waltham, MA, USA). For the NF-κB investigation, some cochlear paraffin sections were obtained, stained for NF-κB using rabbit polyclonal NF-_B p65 (phospho S276) primary antibody (ab194726), goat anti-rabbit IgG H&L (HRP) secondary antibody (ab205718) (Abcam, Cambridge, UK), and 3,3′-diaminobenzidine chromogen following the ABC technique (Ramos-Vara et al. 2008).

### Data analysis

The experimental sample sizes were determined based on relevant research experiences and are listed in the corresponding figure legends and tables footnotes. The SPSS/PC+2001 software was used to analyze existing study results on *n* = 10 independent samples/rats per group size. Data are shown as a means ± the standard error (SE). The data were tested for normality and homogeneity by Shapiro-Wilk *W* test (Shapiro and Wilk 1965) and Levene’s test (O'Neill and Mathews 2002). If the variance was normally distributed and homogenous, one-way ANOVA followed by a post hoc Tukey’s test was used. In addition, the minimum level of significance at *P* < 0.05 was identified. The principal component analysis was conducted using all analysis replicates (Granato et al. 2018).

## Results

### Effects on DOPAE measurements

No significant differences were found between C, OO, and CCM-treated rats in DPOAEs at all tested frequencies (Table 1). In contrast, GEN- and NaS-treated rats showed significant (*P* < 0.001) reductions in DPOAEs at all tested frequencies compared to the control groups. The CCM oral dosing significantly improved the decreased DPOAE responses due to GEN and NaS, particularly at higher frequencies, 6–8 kHz, where non-significant differences were detected compared to the control groups.

**Table 1:** Effect of CCM treatment on the distribution of DPOAE amplitudes for 1–8-kHz frequencies in the ear of GEN or NaS administered rats

Frequencies	Control	OO	CCM	GEN	NaS	CCM+GEN	CCM+NaS
1 kHz	15.03 ± 0.57	15.95 ± 0.97	17.50 ± 1.51	10.72* ± 1.33	12.33* ± 0.26	13.05 ± 0.27	13.93 ± 0.93
2 kHz	16.37 ± 0.58	16.85 ± 0.70	17.17 ± 1.06	10.72* ± 0.65	12.60* ± 0.17	13.43*^#^ ± 0.26	13.55* ± 0.41
3 kHz	14.65 ± 0.66	13.95 ± 0.22	15.83 ± 1.10	11.72* ± 0.48	12.77* ± 0.11	14.23^#^ ± 0.32	14.10 ± 0.35
4 kHz	14.72 ± 0.88	14.53 ± 0.29	15.50 ± 0.81	11.82* ± 0.55	12.42* ± 0.27	14.83^#^ ± 0.54	15.33^#^ ± 0.81
5 kHz	16.92 ± 0.64	16.85 ± 0.65	18.33 ± 0.36	11.53* ± 0.47	12.33* ± 0.37	13.87*^#^ ± 0.76	14.20*^#^ ± 0.65
6 kHz	15.53 ± 0.40	15.47 ± 1.71	16.83 ± 1.12	11.10* ± 0.31	12.12* ± 0.48	14.32^#^ ± 0.61	14.82^#^ ± 0.59
7 kHz	17.82 ± 1.01	16.57 ± 2.38	17.83 ± 0.70	10.58* ± 0.52	11.62* ± 0.21	15.35^#^ ± 0.54	16.07^#^ ± 1.30
8 kHz	17.77 ± 0.63	18.42 ± 1.99	20.00 ± 1.35	11.33* ± 0.42	12.23* ± 0.33	16.67^#^ ± 0.64	18.93^#^ ± 1.10

Values are represented as the mean ± SE. *n* = 10 replicates/treatment

*OO* olive oil

*Significantly different compared to the control group (i.e., CCM, GEN, NaS, GEN+CCM, or CCM+NaS vs. control group) at *P* < 0.05

^#^Significantly different from the respective drug only treated group (i.e., CCM+GEN vs. GEN and CCM+NaS vs. NaS) compared to the control group at *P* < 0.05

### Changes in behavioral performance

#### Effects on the motor coordination

**Fig. 1:**
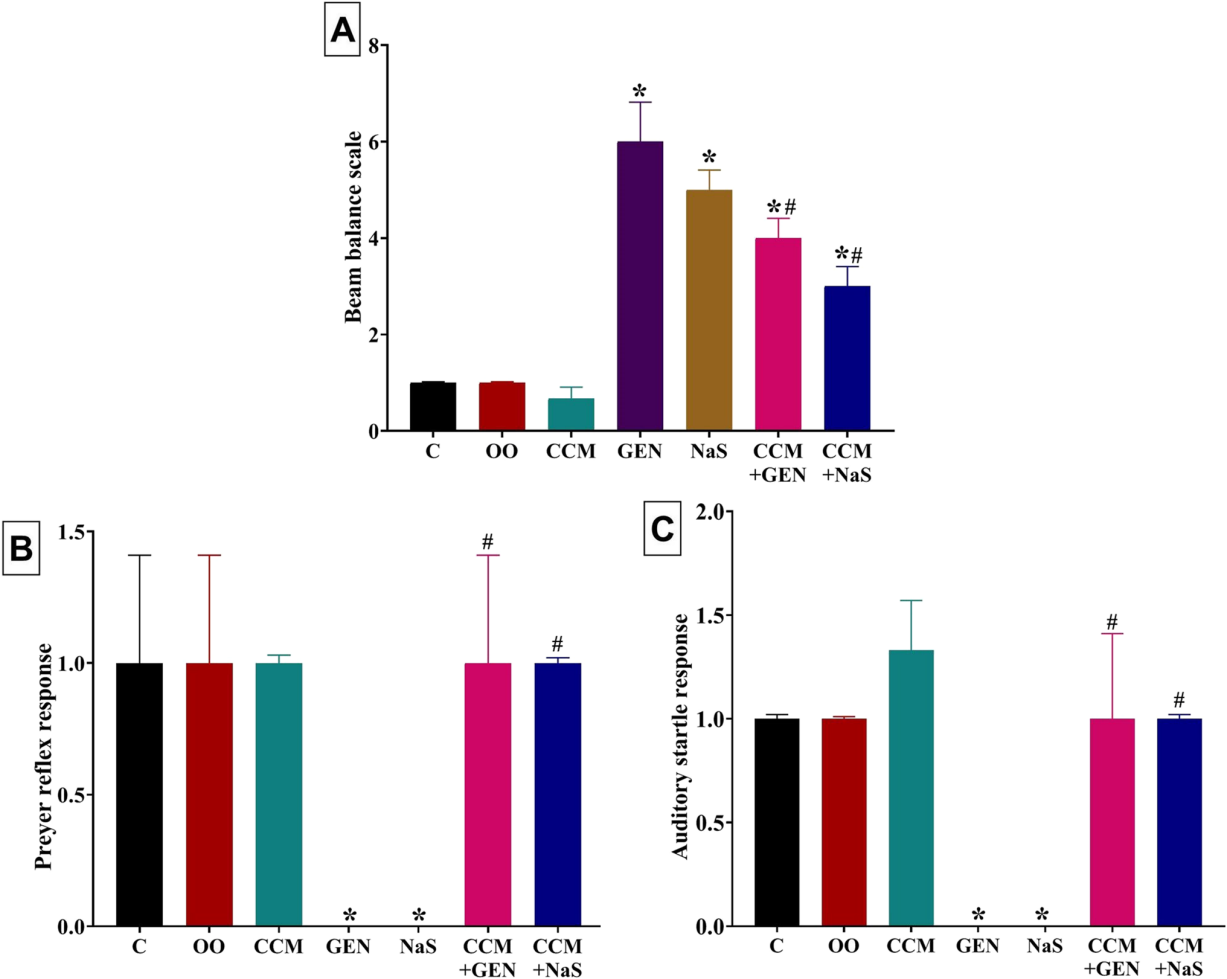
Effect of curcumin (CCM) treatment on **A** the beam balance scale, **B** Preyer reflex response, and **C** auditory startle response of gentamicin (GEN) or sodium salicylate (NaS) administered rats for 15 days. C, control group; OO, olive oil. Data expressed as mean ± SE, *n* = 10 for each group. *Significantly different compared to the control group (i.e., CCM, GEN, NaS, GEN+CCM, or CCM+NaS vs. control group) at *P* < 0.05. ^#^Significantly different from the respective drug-only-treated group (i.e., CCM+GEN vs. GEN and CCM+NaS vs. NaS) compared to the control group at *P* < 0.05

As shown in Fig. 1A, GEN or NaS administration notably impaired motor coordination, denoted by a significant (*P* < 0.001; 500% and 400%, respectively) increase in the beam balance score relative to the control groups. In contrast, CCM oral dosing significantly decreased the beam balance score by 33% in the GEN+CCM group and 40% in the NaS+CCM group compared to the GEN- and NaS-treated groups. Yet, relative to the control group, the beam balance score was still significantly higher by 300% and 200% in the GEN+CCM and NaS+CCM administered rats, respectively.

#### Effects on the hearing

The GEN or NaS injected rats showed hearing deficits demonstrated by significantly lower Preyer reflex (*P* < 0.001) and auditory startle (*P* = 0.018) responses compared to the control group (Fig. 1 B and C). Nonetheless, CCM oral dosing significantly increased the Preyer reflex and auditory startle responses in the GEN+CCM and NaS+CCM groups to the grade that no significant differences exist compared to the control group.

### Effects on serum oxidative stress and lipid peroxidation indicators

**Fig. 2:**
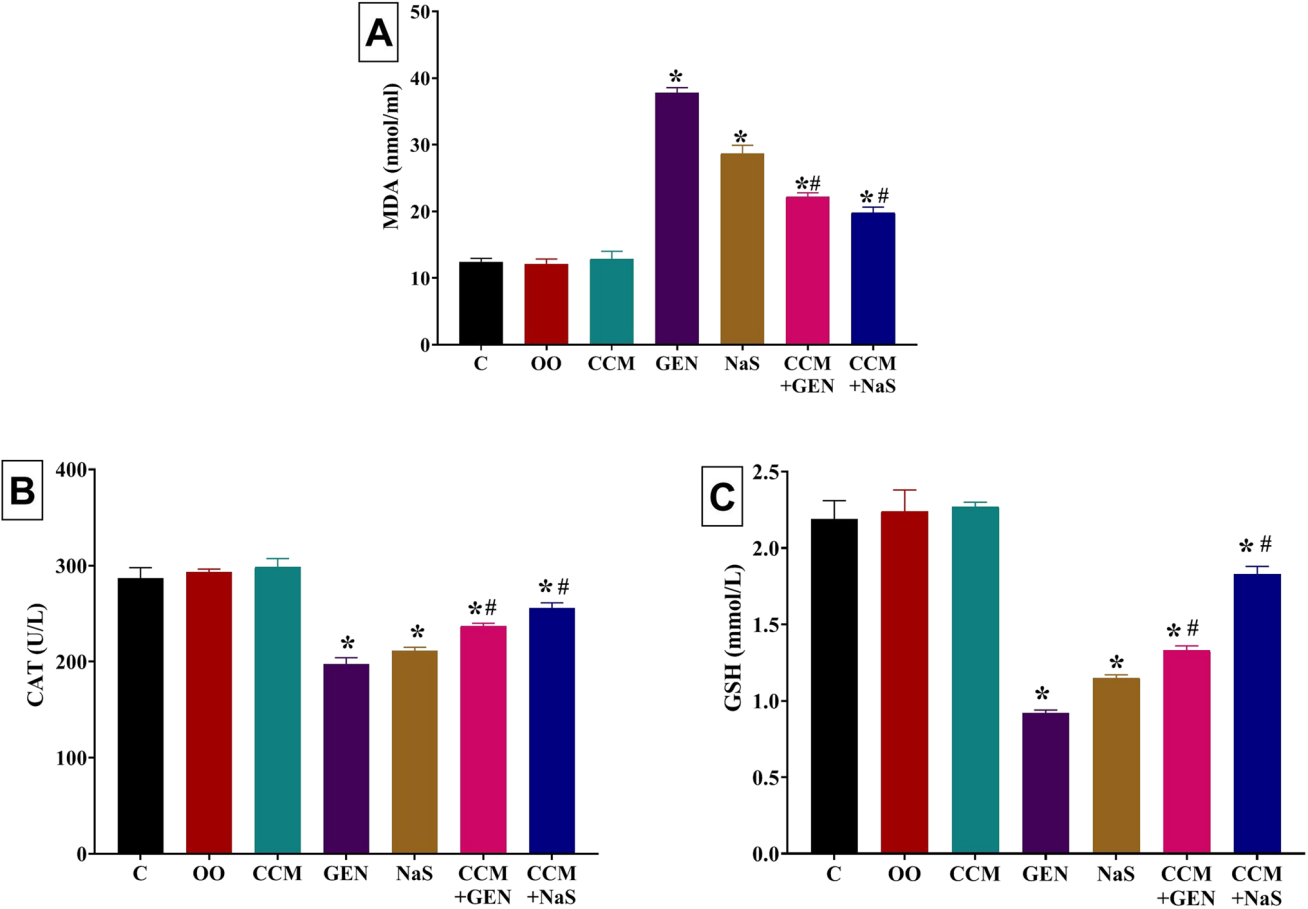
Effect of curcumin (CCM) treatment on serum levels of **A** malondialdehyde (MDA), **B** catalase (CAT), and **C** reduced glutathione (GSH) of gentamicin (GEN) or sodium salicylate (NaS) administered rats for 15 days. C, control group; OO, olive oil. Data expressed as mean ± SE, *n* = 10 for each group. *Significantly different from the control group (i.e., CCM, GEN, NaS, GEN+CCM, or CCM+NaS vs. control group) at *P* < 0.05. ^#^Significantly different from the respective drug-only-treated group (i.e., CCM+GEN vs. GEN and CCM+NaS vs. NaS) compared to the control group at *P* < 0.05

Significant increases in the MDA level were recorded in GEN (204%)- or NaS (131%)-treated rats relative to the control group (Fig. 2A). Yet, CCM significantly reduced the MDA level by 41% and 31% in CCM+GEN- and CCM+NaS-treated groups, respectively, relative to GEN- and NaS-treated groups. Nevertheless, relative to the control group, the MDA level was still significantly high at 79% and 59% in CCM+GEN- and CCM+NaS-treated groups, respectively. As shown in Fig. 2B and C, apparent (*P* < 0.001) exhaustion of enzymatic antioxidant, CAT (31% and 27%, respectively), and non-enzymatic antioxidant, GSH (58% and 48% reduction, respectively), were noted in GEN- and NaS-treated rats, respectively, compared to the control group. In contrast, the CAT and GSH contents were significantly increased by 20% and 21%, respectively, in CCM+GEN- than GEN-treated group and by 45% and 60%, respectively, in CCM+NaS- than NaS-treated group. Nonetheless, relative to the control group, the CAT and GSH contents were still significantly lower by 17% and 11%, respectively, in CCM+GEN and 39% and 16%, respectively, in CCM+NaS-treated group.

## Histopathological findings

**Fig. 3:**
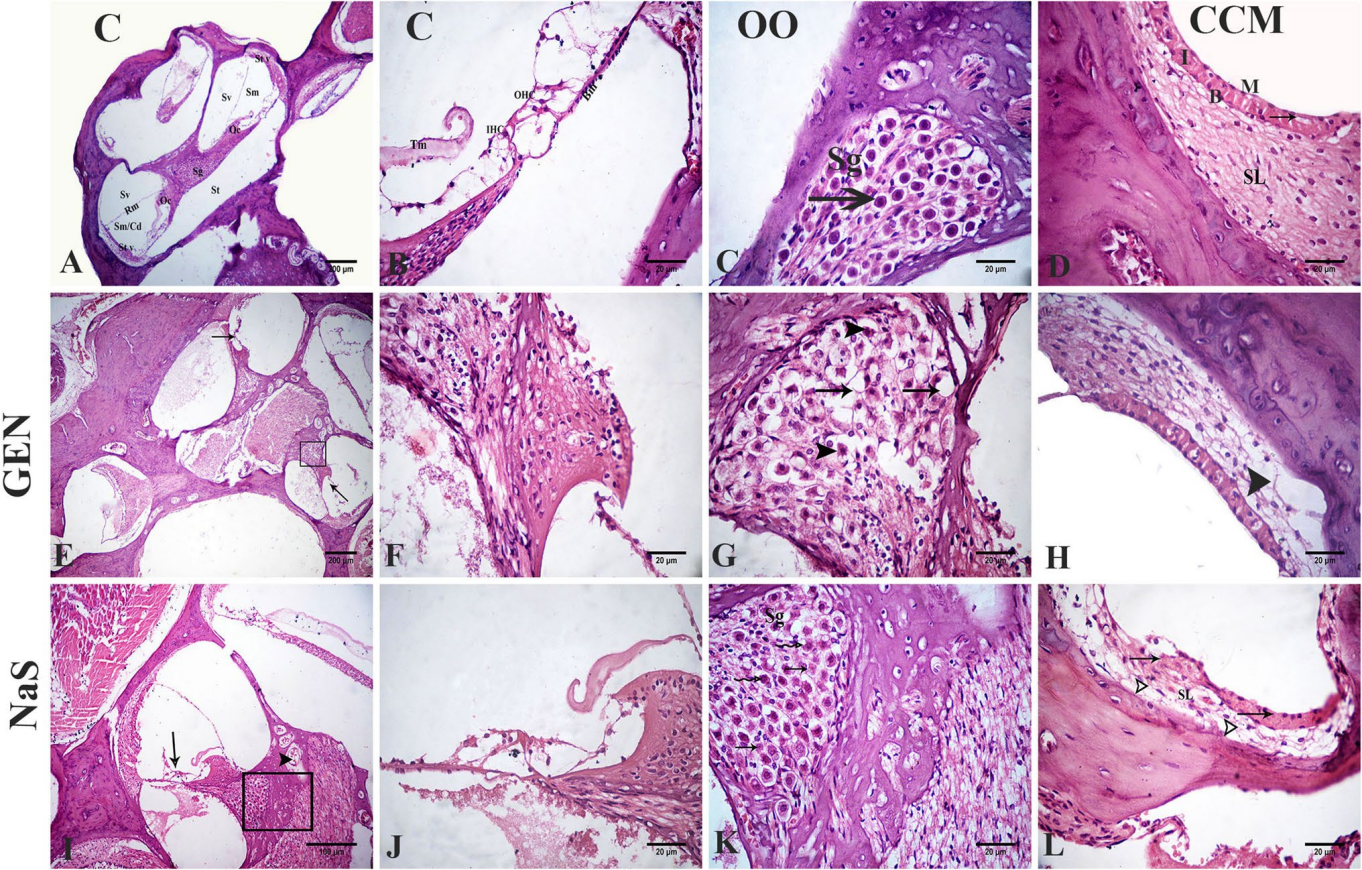
Photomicrograph of H&E-stained sections of the rat inner ear. Control (**A**, **B**), olive oil (**C**), curcumin (**D**), GEN (**E**–**H**), and NaS treated groups (**I**–**L**). The cochlear cavity is composed of three fluids-filled spaces: the scala vestibuli (Sv), scala media (Sm) or cochlear duct (Cd), and the scala tympani (St) and contains the Reissener’s membrane (Rm). The organ of Corti (Oc) that bounded laterally by the stria vascularis (St v) and medially by the spiral ganglion (Sg) (**A**). The higher magnification of the cellular element of the organ of Corti contained the tectorial membrane (Tm), inner hair cell (IHC) and 3 outer hair cells (OHC), and the intact basilar membrane (Bm) (**B**). The spiral ganglion (Sg), is situated inside the modiolus and contains neurons (arrow) of the cochlear nerve (**C**). The stria vascularis shows the intraepithelial capillary (arrow), and the three layers of cells, marginal cells (M), intermediate cells (I), basal cells (B), and the spiral ligament (SL) were formed of a connective tissue containing fibrocytes (**D**). Loss of hair cells in the organ of Corti (arrows) (**E**). The higher magnification of the organ of Corti with loss of hair cells (**F**). The higher magnification of the spiral ganglion with degeneration and necrosis in spiral ganglion cells (arrowheads) with the presence of large vacuoles (arrows) (**G**). Vacuolation in the spiral ligament (arrowhead) (**H**). Decreased number of hair cells (arrow) in the organ of Corti and congested blood vessels (arrowhead) (**I**). The higher magnification of the organ of Corti with decreased number of hair cells (**J**). The higher magnification of the spiral ganglion (Sg) with shrinked neurons (arrows) and necrotic neurons (zigzag arrows) (**K**). The stria vascularis with cracking in the epithelium (arrows). Spiral ligament (SL) with vacuolation (arrowheads) (**L**)

The control, OO, and CCM groups showed normal histological cochlea architecture. The organ of Corti was located inside the scala media, on the basal membrane. This structure contained internal and external sensory hair cells. The vestibular membrane separates scala vestibuli and scala media. Normally, the tectorial membrane is settled on Corti organ hair cells. The stria vascularis and the spiral ligament are inside the lateral wall’s scale media; the ligament was formed of connective tissue containing fibrocytes. The stria vascularis consisted of three layers of cells, marginal cells, intermediate cells, and basal cells. The spiral ganglion was embedded within the modiolus and contained the cochlear nerve neurons (Fig. 3A–D).

In GEN-treated rats, a severe loss in the hair cells in Corti’s organ was evident. The spiral ganglion neurons showed necrosis and appeared shrunken with deeply stained nuclei with the presence of large vacuoles in the spiral ligament (Fig. 3E–H). In NaS-treated rats, a decreased number of hair cells in the organ of Corti and congested blood vessels was recorded (Fig. 3I and J). Many sections of spiral ganglion neurons showed degeneration and disorganized stria vascularis epithelium (Fig. 3K). Congested intraepithelial blood capillaries were also seen. Highly vacuolated fibrocytes of the spiral ligament were detected (Fig. 3L).

**Fig. 4:**
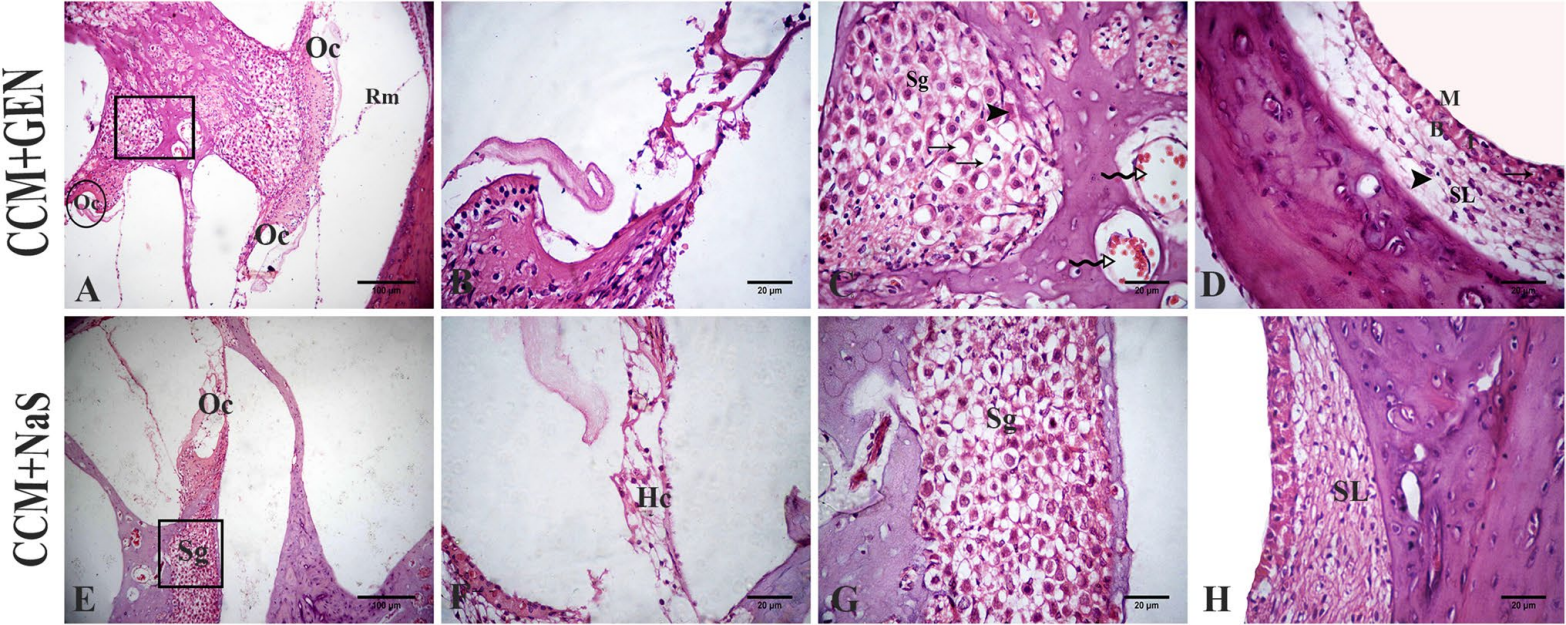
Photomicrograph of H&E-stained sections of the rat inner ear. CCM+GEN-treated groups (**A**–**D**). CCM+NaS-treated groups (**E**–**H**). Organ of Corti (Oc) with decreased hair cells, spiral ganglia (Sg), and Reissener’s membrane (Rm) (**A**). The higher magnification of the circle of **A** represent organ of Corti (**B**). The higher magnification to the spiral ganglion (Sg) of **A** showed a few hypereosinophilic neurons (arrowhead), congested blood vessels (zigzag arrows), and some vacuoles (arrows) (**C**). *Stria vascularis* with three layers of cells, marginal cells (M), intermediate cells (I), and basal cells (B), showed mild congestion in the vessels (arrow), also the spiral ligament (SL) with mild vacuolation (arrowhead) (**D**). Organ of Corti and spiral ganglion of NaS+CCM-treated groups (**E**). Higher magnification to the organ of Corti with its hair cells (HC) and spiral ganglion in **F** and **G**. The *stria vascularis* and the spiral ligament (SL) appeared normal (**H**)

In the GEN+CCM group, a moderate decrease was observed in outer hair cell numbers in the organ of Corti and congested blood vessels. Mild degeneration was determined in spiral ganglion cells with vacuolation (Fig. 4A–C). There were mild dilatation and congestion in the vessels of the stria vascularis, and some intermediate cells appeared vacuolated. Mild vacuolated fibrocytes of the spiral ligament were detected (Fig. 4D). In NaS+CCM-treated rats, a mild reduction in the number of hair cells was observed in the organ of Corti. Mild degenerated neurons were also detected in spiral ganglion cells. The spiral ligament appeared without vacuolation, and the cells of stria vascularis were normally organized (Fig. 4E–H). The lesion scoring of all experimental groups’ cochlear structures of the ear was scored in Table 2.

**Table 2:** Effect of CCM treatment on lesion scoring of the cochlear structures of the ear of GEN or NaS administered rats

Lesions	Control	OO	CCM	GEN	NaS	CCM+GEN	CCM+NaS
Spiral ganglion							
Necrotic neurons	0.00 ± 0.00	0.00 ± 0.00	0.00 ± 0.00	3.17* ± 0.26	3.50* ± 0.29	1.67*^#^ ± 0.36	1.83*^#^ ± 0.51
Vacuolation	0.17 ± 0.14	0.17 ± 0.14	0.33 ± 0.18	2.67* ± 0.18	1.17* ± 0.34	1.17*^#^ ± 0.26	0.83 ± 0.26
Decreased number of spiral ganglion cells	0.00 ± 0.00	0.00 ± 0.00	0.00 ± 0.00	2.17* ± 0.34	1.83* ± 0.26	1.17*^#^ ± 0.14	1.00*^#^ ± 0.22
Stria vascularis							
Cracking of epithelium	0.67 ± 0.28	0.00 ± 0.00	0.00 ± 0.00	0.83* ± 0.26	2.83* ± 0.34	0.50 ± 0.29	1.17*^#^ ± 0.40
Congested capillaries	0.00 ± 0.00	0.67 ± 0.18	0.67 ± 0.18	1.00 ± 0.00	2.05* ± 0.34	1.00 ± 0.31	1.50* ± 0.29
Organ of Corti							
Loss of hair cells	0.00±0.00	0.00 ± 0.00	0.00 ± 0.00	4.17* ± 0.34	2.83* ± 0.34	3.00*^#^ ± 0.22	1.83*^#^ ± 0.26

Values are represented as the mean ± SE. *n* = 10 replicates/treatment

*OO* olive oil

*Significantly different compared to the control group (i.e., GEN, NaS, GEN+CCM, or CCM+NaS vs. control group) at *P* < 0.05

^#^Significantly different from the respective drug only treated group (i.e., CCM+GEN vs. GEN and CCM+NaS vs. NaS) compared to the control group at *P* < 0.05

## Immunohistochemical findings

**Fig. 5:**
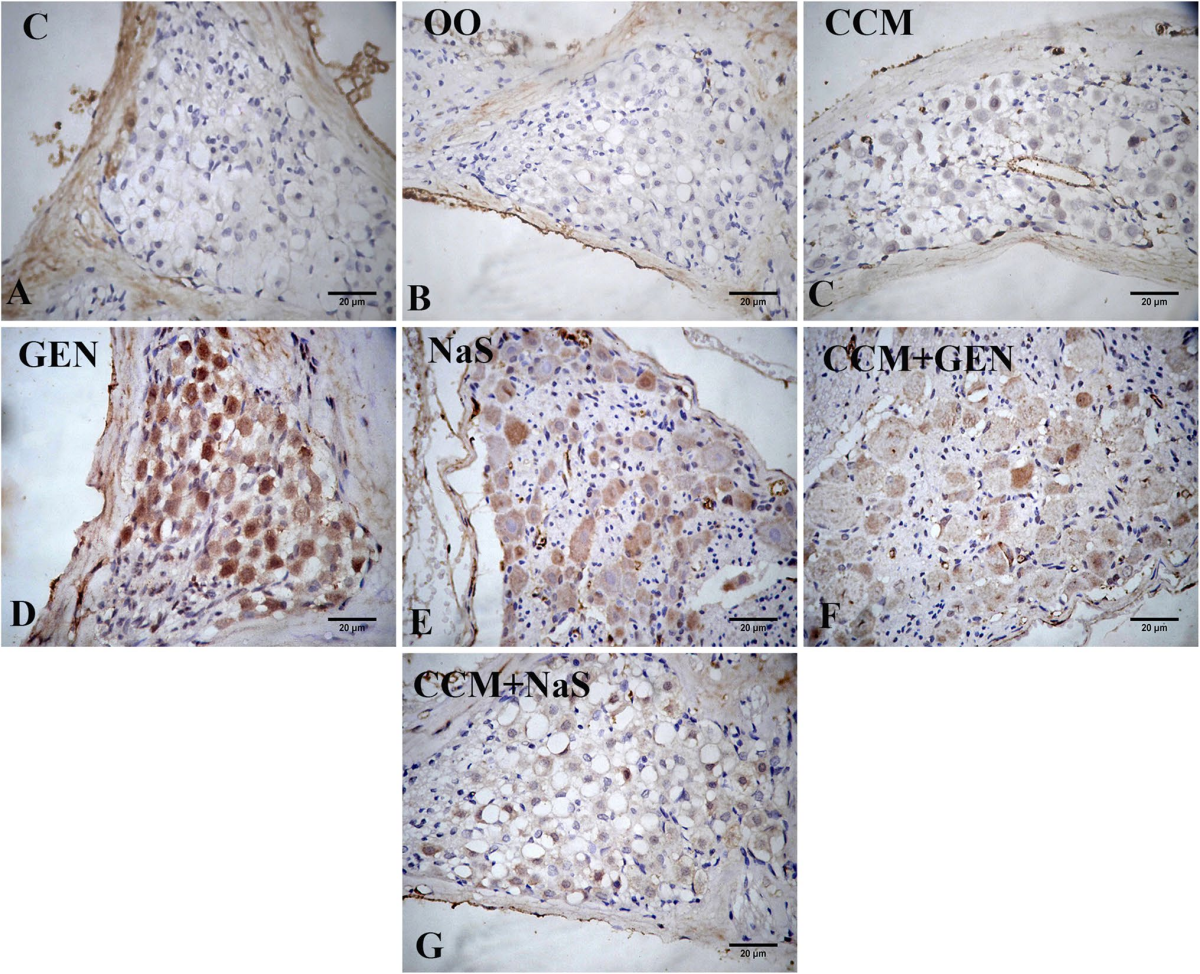
Photomicrograph of the spiral ganglia of inner ear rat tissue sections showing the immunoexpression of caspase-3 as follows: negative in control (**A**), olive oil (**B**), and curcumin (CCM)-treated rats (**C**), strong in the gentamicin (GEN) (**D**), moderate in sodium salicylate (NaS) (**E**), mild in the GEN+CCM-treated rats (**F**) and weak in NaS+CCM-treated rats (**G**)

**Fig. 6:**
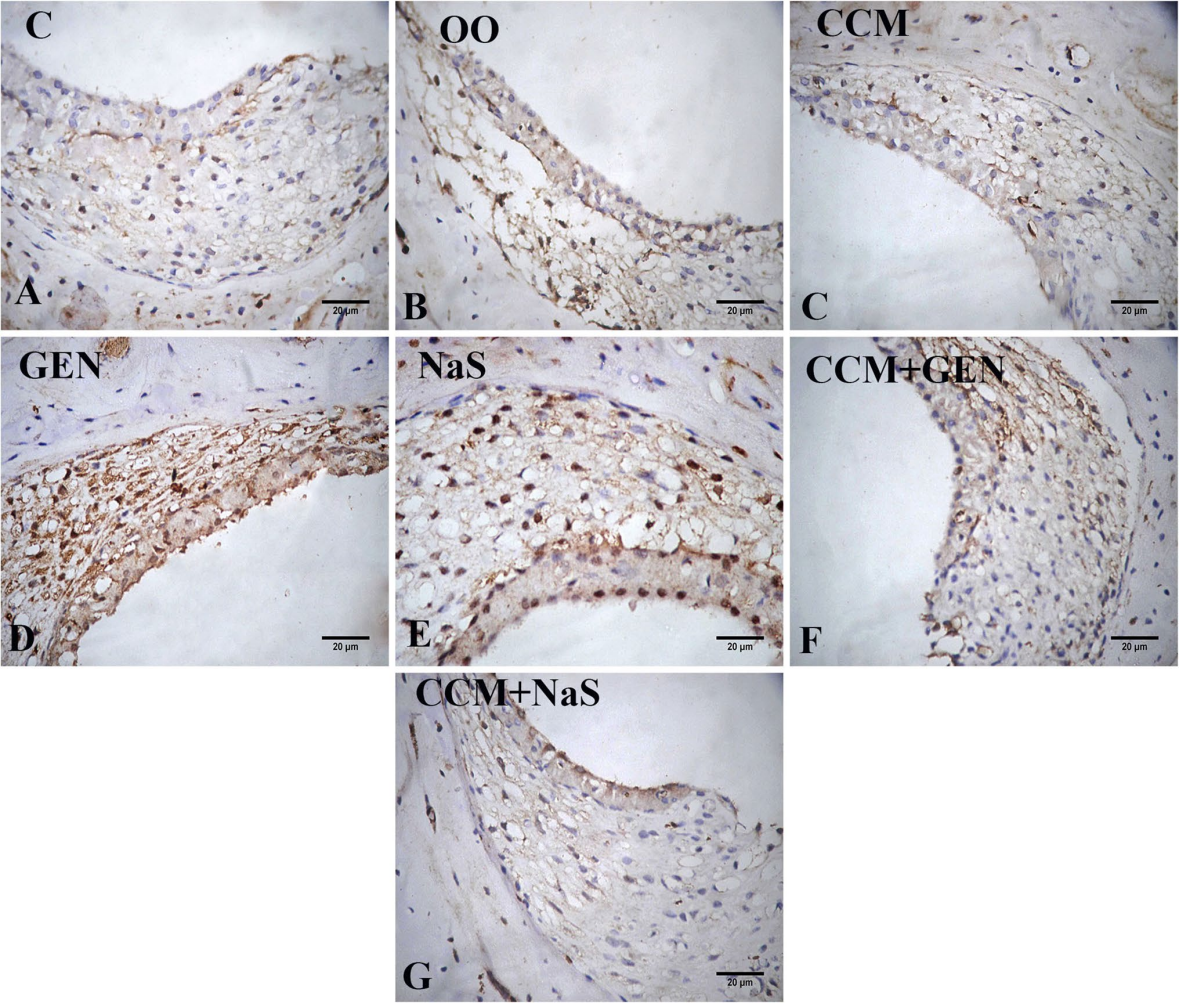
Photomicrograph of the *stria vascularis* of inner ear rat tissue sections showing the immunoexpression of NF-κB in control (**A**), olive oil (**B**), and curcumin (CCM) (**C**), gentamicin (GEN) (**D**), sodium salicylate (NaS) (**E**), GEN+CCM (**F**), and NaS+CCM (**G**) treated rats

**Fig. 7:**
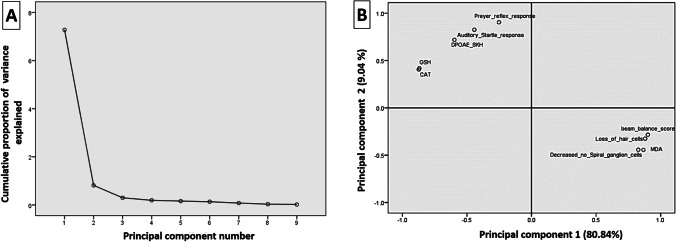
The principal component analysis plot shows the estimated variables’ relationships. **A** Cumulative variance proportion is a function of the number of principal components (PC). **B** All biochemical and neurobehavioral indicators plotted as a function of PC1 and PC2, which account for 80.84% and 9.04% of the variance, respectively. GSH, reduced glutathione; CAT, catalase; MDA, malondialdehyde; DPOAE, distortion product otoacoustic emission

The caspase-3 immunoexpression was negative in the spiral ganglia in control (Fig. 5A), olive oil (Fig. 5B), and CCM-treated groups (Fig. 5C). The GEN and NaS injection for 15 days upregulated the expression of caspase-3 tissue in the spiral ganglia (Fig. 5D and E) compared to the control group. In concurrently treated groups, the expression of caspase-3 was downregulated (Fig. 5F and G). The immunoexpression of NF-κB was studied in the *stria vascularis*. In control, olive oil, and CCM-treated groups, a weak NF-κB positive reaction in the cytoplasm of fibrocyte of spiral ligament and a negative reaction of epithelial layers of *stria vascularis* were recorded (Fig. 6A, B, C). In GEN-treated rats, a strong positive reaction was observed in the spiral ligament nuclei (Fig. 6D). In the NaS-treated group, the positive reaction decreased compared to GEN group (Fig. 7E). The downregulation of the NF-κB expression in NaS+CCM-treated rats was more evident than in the GEN+CCM-treated rats (Fig. 6F, G).

## Principal components analysis findings

The principal component analysis examined the correlations between the current variables in the present study (Fig. 7A). As shown in Fig. 7B, the loading plot of the first two components was shown, and both components constituted nearly 89.88% of the overall variation in the trial data. In the loading map, the closely grouped variables (< 90°) have good correlations and are positively associated, as well as vice versa. Consequently, MDA, beam balance score, decreased number of spiral ganglia, and loss of hair cells clustered together and strongly correlated with the first component. These clusters were negatively correlated with Preyer reflex response, auditory startle response, CAT, GSH, and DPOAE responses at the highest frequencies (8 kHz).

## Discussion

In the present study, significant reductions in auditory function as reduced DOPAE measurements were apparent in GEN- or NaS-treated rats compared to the control group. The impaired hearing function was reflected in the behavioral findings in the auditory startle response, Preyer reflex, and beam balance scale. Numerous pathological alterations were observed in the inner ear tissues of the GEN- or NaS-injected rats comprising severe loss in the Corti organ’s hair cells, degenerated spiral ganglion neurons, atrophied vestibular ganglion, and congestion of intraepithelial blood capillaries. The former damages suggest that various inner area defects may be responsible for impaired balance and weakened hearing function. Comparably, Park et al. (2017) demonstrated that GEN significantly reduced hair cell numbers in the organ of Corti explants. Also, in the recent study by Kim et al. (2021), the intratympanic GEN injection in rats resulted in a near-complete loss of hair cells and a collapse of the sensory epithelium in both the saccule and utricle. NaS has also been found to cross the blood–brain barrier and interfere directly with neuronal activity at locations in the central auditory system (Chen et al. 2013; Eggermont 2015). Additionally, the NaS-induced ototoxicity may also comprise blood circulation disorder in the inner ear since these drugs can cause vasoconstriction and decrease cochlear blood flow (Didier et al. 1993). Besides, the disturbing neural output of the cochlea could partly be implicated in their dysfunction. In this regard, NaS and GEN evoked evident neurotoxic effects reflected in neurobehavioral aberrations and depleted gamma-aminobutyric acid (GABA) neurotransmitter content in our earlier work (Abd-Elhakim et al. 2021a).

However, CCM was principally effective in maintaining the hearing function, reflected by a significant improvement in DOPAE at most tested frequencies even with GEN or NaS administration. Similar findings were previously recorded in the studies of Soyalıç et al. (2016) and Soyalıç et al. (2017). In this regard, CCM has been identified as vasodilatory and used to lower blood pressure, benefitting blood circulation to Corti’s organ (Nugroho et al. 2008). CCM-mediated brain neurotransmitter restoration may also be part of a possible complex cascade of events, contributing to improved hearing and balance efficiency (Bhutani et al. 2009).

Cumulative evidence verified the critical role of oxidative stress and apoptosis in GEN- and NaS-induced ototoxic effects (Park et al. 2017; Stypulkowski 1990). Herein, GEN and NaS considerably depleted CAT activity and GSH content but raised serum MDA and caspase-3 immunoexpression in inner ear tissue. In vitro and in vivo studies, GEN has been shown to enhance ROS generation and promote apoptotic events (Bustos et al. 2016; Bustos et al. 2018; Mohamed et al. 2019a). Also, GEN has been reported to deplete the antioxidant activity (Haryuna et al. 2017) but increase the apoptotic index (Haryuna et al. 2018) in the lateral wall of the cochlea fibroblasts. Despite the distinguished antioxidant effect of low doses of NaS (Yiannakopoulou and Tiligada 2009), NaS can be a prooxidant that promotes cell death at high doses. Deng et al. (2013) have documented that high NaS levels led to radical superoxide upregulation and apoptosis of spiral ganglion neurons in vitro. NaS-induced apoptosis was also demonstrated by p38-activated mitogen protein kinases leading to the Caspase-3 activation (Lee et al. 2003). The oxidative and lipid peroxidative damage and apoptotic activity caused by GEN and NaS thus elucidate the loss of hair cells detected during cochlear histopathology.

The CCM otoprotective ability may be closely related to its potent antioxidant and antiapoptotic activities. Correspondingly, CCM usage decreased cellular apoptosis and had a profound safety effect on auditory function in an acoustic trauma rat model (Soyalıç et al. 2017). CCM also stopped caspase-3 activation, modifying the expression of the Bcl-2 family in spiral ganglion neurons activated by peroxynitrite (Liu et al. 2011). Moreover, CCM significantly counteracted the apoptotic events resulting from intratympanic injection of GEN for 18 h and diabetes mellitus-induced oxidative stress in the lateral wall of the cochlea fibroblasts (Haryuna et al. 2017; Haryuna et al. 2018). One or more interactions may be part of the proposed antioxidant mechanism of CCM, like neutralizing or scavenging free radicals, preventing oxidative cascades, quenching oxygen, inhibiting oxidative enzymes, and deactivating toxicants oxidative properties (Rao 1994; Unnikrishnan and Rao 1995). Moreover, CCM's antioxidant action is closely tied to its conjugating structure, consisting of two methoxylated phenols and an enol diketone that traps radicals (Masuda et al. 2001). Besides, CCM therapy stimulates detoxification enzymes due to free radical scavenging and lysosomal release inhibition (Manikandan et al. 2004). In addition, CCM helps to protect the integrity of the cell membrane when there are toxicants by peroxidation prevention (Sankar et al. 2012).

Herein, an apparent increased NF-κB immunoexpression in the inner ear tissue following GEN or NaS administration was detected but suppressed following CCM treatment. Comparably, several NF-κB activation reports have been recorded in the cochlea after stress, including acoustic exposure (Miyao et al. 2008; Selivanova et al. 2007), administration of ototoxic drugs (Jiang et al. 2005; Watanabe et al. 2002), and inflammatory challenges (Moon et al. 2007). Also, Adams et al. (2009) reported that intense noise exposure elicited NF-κB activation in the mouse inner ear cells. Previous research has indicated that NF-κB pathways play a role in GEN toxicity (Ozbek et al. 2009; Volpini et al. 2006). NF-κB is inactive in the cytoplasm of resting cells due to binding with its inhibitors, p105 and inhibitor kB-alpha (I kB -alpha) like proteins (Baldwin Jr 1996). The increased ROS secondary to GEN therapy causes the proteolytic cleavage of p105 or the degradation of IkB-alpha and consequently free NF-κB dimers translocates to nucleus and upregulated (Ozbek et al. 2009). On the other hand, Hoppstädter et al. (2016) reported that the CCM anti-inflammatory activities are based on the modulation of transcribed factors, growth factors, signal transduction pathways, and inflammatory cytokines via inhibiting NF-κB signaling.

The principal component analysis is a popular multivariate statistical approach since it separates samples using a two-dimensional projection (Granato et al. 2018). The loading plot results showed a strong correlation between the measured parameter responses. A strong positive correlation exists between hearing function indicators and antioxidants level in this study. Correspondingly, numerous previous studies have demonstrated that endogenous antioxidant deficiency is significantly associated with hearing loss (Seidman et al. 2002; Tavanai and Mohammadkhani 2017).

## Conclusion

Overall, the current study’s combined audiological, behavioral, biochemical, histopathological, and immunohistochemical results propose that the GEN and NaS exposure at high dosages could be ototoxic. The GEN and NaS induced reduced DPOAE deficit hearing functions, impaired balance, and altered inner ear architecture might be mediated mostly through oxidative damage, apoptotic changes, and NF-κB activation. Moreover, CCM could be a prospective protective nominee of GEN and NaS accompanied ototoxic effect through controlling apoptotic and inflammatory pathways through antioxidant properties.

## Data Availability

The datasets used and/or analyzed during the current study are available from the corresponding author on reasonable request.

## Abbreviations

CAT: catalase
CCM: curcumin
dB: decibels
DP: DPOAE plot
DPOAE: distortion product otoacoustic emission
GEN: gentamicin
GSH: reduced glutathione
MDA: malondialdehyde
NaS: sodium salicylates
NF-κB: nuclear factor-kappa
OO: olive oil
